# Supplementary material for: AffyMAPSDetector: a software tool to characterize Affymetrix GeneChip™ expression arrays with respect to SNPs
Source: BMC Bioinformatics. 2007 Jul 30;8:276. doi: 10.1186/1471-2105-8-276 (PMC1959249; doi:10.1186/1471-2105-8-276)
Supplement: Additional file 13 — AffyMAPSDetector v1 source code. AffyMAPSDetector source code. [file 1471-2105-8-276-S13.zip › AffyMAPSDetector_v1_src/doc/affychip/snpdetector/ui/StepTwoPanel.html]

StepTwoPanel


|  |  |  |  |
| --- | --- | --- | --- |
| **Overview** \| **Package** \| **Class** \| **Use** \| **Tree** \| **Deprecated** \| **Index** \| **Help** | | |  |
| **PREV CLASS**   NEXT CLASS | **FRAMES**    **NO FRAMES**     **All Classes** |  |  |
| SUMMARY: NESTED \| FIELD \| CONSTR \| METHOD | DETAIL: FIELD \| CONSTR \| METHOD |  |  |


---


## affychip.snpdetector.ui Class StepTwoPanel

```
java.lang.Object
  java.awt.Component
      java.awt.Container
          javax.swing.JComponent
              javax.swing.JPanel
                  jwf.WizardPanel
                      affychip.snpdetector.ui.StepTwoPanel
```

**All Implemented Interfaces:**: javax.accessibility.Accessible, java.awt.image.ImageObserver, java.awt.MenuContainer, java.io.Serializable

---

public class **StepTwoPanel** extends WizardPanel

Title: Probe SNP Detector

Description: This class represents 2nd step of FileLoaderWizard.
It prompts the user to load Probeset Information File and gathers the info
such as indices of the columns corresponding to Probeset ID (i.e. name),
Probe's X Position, Probe's Y Position, Interrogation Position, and
Probe Sequence.

Copyright: Sunita Kumari -- Copyright (c) 2004

Company: George Mason University

**Version:**
:   1.1

**Author:**
:   Sunita Kumari

**See Also:**: Serialized Form

---

**Nested Class Summary**

**Nested classes inherited from class javax.swing.JPanel**

`javax.swing.JPanel.AccessibleJPanel`

**Nested classes inherited from class javax.swing.JComponent**

`javax.swing.JComponent.AccessibleJComponent`

**Nested classes inherited from class java.awt.Container**

`java.awt.Container.AccessibleAWTContainer`

**Nested classes inherited from class java.awt.Component**

`java.awt.Component.AccessibleAWTComponent, java.awt.Component.BltBufferStrategy, java.awt.Component.FlipBufferStrategy`


**Field Summary**

**Fields inherited from class jwf.WizardPanel**

`wizardContext`

**Fields inherited from class javax.swing.JComponent**

`accessibleContext, listenerList, TOOL_TIP_TEXT_KEY, ui, UNDEFINED_CONDITION, WHEN_ANCESTOR_OF_FOCUSED_COMPONENT, WHEN_FOCUSED, WHEN_IN_FOCUSED_WINDOW`

**Fields inherited from class java.awt.Component**

`BOTTOM_ALIGNMENT, CENTER_ALIGNMENT, LEFT_ALIGNMENT, RIGHT_ALIGNMENT, TOP_ALIGNMENT`

**Fields inherited from interface java.awt.image.ImageObserver**

`ABORT, ALLBITS, ERROR, FRAMEBITS, HEIGHT, PROPERTIES, SOMEBITS, WIDTH`


**Constructor Summary**

`StepTwoPanel()`


|  |  |
| --- | --- |
| **Method Summary** | |
| `boolean` | `canFinish()`             Can this panel finish the wizard? |
| `void` | `display()`             Called when the panel is set. |
| `void` | `finish()`             Handle finishing the wizard. |
| `boolean` | `hasNext()`             Is there be a next panel? |
| `WizardPanel` | `next()`             Get the next panel to go to. |
| `boolean` | `validateFinish(java.util.List list)`             Called to validate the panel before finishing the wizard. |
| `boolean` | `validateNext(java.util.List list)`             Called to validate the panel before moving to next panel. |

**Methods inherited from class jwf.WizardPanel**

`getWizardContext, hasHelp, help, setWizardContext`

**Methods inherited from class javax.swing.JPanel**

`getAccessibleContext, getUI, getUIClassID, paramString, setUI, updateUI`

**Methods inherited from class javax.swing.JComponent**

`addAncestorListener, addNotify, addPropertyChangeListener, addPropertyChangeListener, addVetoableChangeListener, computeVisibleRect, contains, createToolTip, disable, enable, firePropertyChange, firePropertyChange, firePropertyChange, firePropertyChange, firePropertyChange, firePropertyChange, firePropertyChange, firePropertyChange, firePropertyChange, fireVetoableChange, getActionForKeyStroke, getActionMap, getAlignmentX, getAlignmentY, getAncestorListeners, getAutoscrolls, getBorder, getBounds, getClientProperty, getComponentGraphics, getConditionForKeyStroke, getDebugGraphicsOptions, getDefaultLocale, getGraphics, getHeight, getInputMap, getInputMap, getInputVerifier, getInsets, getInsets, getListeners, getLocation, getMaximumSize, getMinimumSize, getNextFocusableComponent, getPreferredSize, getPropertyChangeListeners, getPropertyChangeListeners, getRegisteredKeyStrokes, getRootPane, getSize, getToolTipLocation, getToolTipText, getToolTipText, getTopLevelAncestor, getTransferHandler, getVerifyInputWhenFocusTarget, getVetoableChangeListeners, getVisibleRect, getWidth, getX, getY, grabFocus, isDoubleBuffered, isLightweightComponent, isManagingFocus, isMaximumSizeSet, isMinimumSizeSet, isOpaque, isOptimizedDrawingEnabled, isPaintingTile, isPreferredSizeSet, isRequestFocusEnabled, isValidateRoot, paint, paintBorder, paintChildren, paintComponent, paintImmediately, paintImmediately, print, printAll, printBorder, printChildren, printComponent, processComponentKeyEvent, processKeyBinding, processKeyEvent, processMouseMotionEvent, putClientProperty, registerKeyboardAction, registerKeyboardAction, removeAncestorListener, removeNotify, removePropertyChangeListener, removePropertyChangeListener, removeVetoableChangeListener, repaint, repaint, requestDefaultFocus, requestFocus, requestFocus, requestFocusInWindow, requestFocusInWindow, resetKeyboardActions, reshape, revalidate, scrollRectToVisible, setActionMap, setAlignmentX, setAlignmentY, setAutoscrolls, setBackground, setBorder, setDebugGraphicsOptions, setDefaultLocale, setDoubleBuffered, setEnabled, setFont, setForeground, setInputMap, setInputVerifier, setMaximumSize, setMinimumSize, setNextFocusableComponent, setOpaque, setPreferredSize, setRequestFocusEnabled, setToolTipText, setTransferHandler, setUI, setVerifyInputWhenFocusTarget, setVisible, unregisterKeyboardAction, update`

**Methods inherited from class java.awt.Container**

`add, add, add, add, add, addContainerListener, addImpl, applyComponentOrientation, areFocusTraversalKeysSet, countComponents, deliverEvent, doLayout, findComponentAt, findComponentAt, getComponent, getComponentAt, getComponentAt, getComponentCount, getComponents, getContainerListeners, getFocusTraversalKeys, getFocusTraversalPolicy, getLayout, insets, invalidate, isAncestorOf, isFocusCycleRoot, isFocusCycleRoot, isFocusTraversalPolicySet, layout, list, list, locate, minimumSize, paintComponents, preferredSize, printComponents, processContainerEvent, processEvent, remove, remove, removeAll, removeContainerListener, setFocusCycleRoot, setFocusTraversalKeys, setFocusTraversalPolicy, setLayout, transferFocusBackward, transferFocusDownCycle, validate, validateTree`

**Methods inherited from class java.awt.Component**

`action, add, addComponentListener, addFocusListener, addHierarchyBoundsListener, addHierarchyListener, addInputMethodListener, addKeyListener, addMouseListener, addMouseMotionListener, addMouseWheelListener, bounds, checkImage, checkImage, coalesceEvents, contains, createImage, createImage, createVolatileImage, createVolatileImage, disableEvents, dispatchEvent, enable, enableEvents, enableInputMethods, getBackground, getBounds, getColorModel, getComponentListeners, getComponentOrientation, getCursor, getDropTarget, getFocusCycleRootAncestor, getFocusListeners, getFocusTraversalKeysEnabled, getFont, getFontMetrics, getForeground, getGraphicsConfiguration, getHierarchyBoundsListeners, getHierarchyListeners, getIgnoreRepaint, getInputContext, getInputMethodListeners, getInputMethodRequests, getKeyListeners, getLocale, getLocation, getLocationOnScreen, getMouseListeners, getMouseMotionListeners, getMouseWheelListeners, getName, getParent, getPeer, getSize, getToolkit, getTreeLock, gotFocus, handleEvent, hasFocus, hide, imageUpdate, inside, isBackgroundSet, isCursorSet, isDisplayable, isEnabled, isFocusable, isFocusOwner, isFocusTraversable, isFontSet, isForegroundSet, isLightweight, isShowing, isValid, isVisible, keyDown, keyUp, list, list, list, location, lostFocus, mouseDown, mouseDrag, mouseEnter, mouseExit, mouseMove, mouseUp, move, nextFocus, paintAll, postEvent, prepareImage, prepareImage, processComponentEvent, processFocusEvent, processHierarchyBoundsEvent, processHierarchyEvent, processInputMethodEvent, processMouseEvent, processMouseWheelEvent, remove, removeComponentListener, removeFocusListener, removeHierarchyBoundsListener, removeHierarchyListener, removeInputMethodListener, removeKeyListener, removeMouseListener, removeMouseMotionListener, removeMouseWheelListener, repaint, repaint, repaint, resize, resize, setBounds, setBounds, setComponentOrientation, setCursor, setDropTarget, setFocusable, setFocusTraversalKeysEnabled, setIgnoreRepaint, setLocale, setLocation, setLocation, setName, setSize, setSize, show, show, size, toString, transferFocus, transferFocusUpCycle`

**Methods inherited from class java.lang.Object**

`clone, equals, finalize, getClass, hashCode, notify, notifyAll, wait, wait, wait`

**Constructor Detail**

### StepTwoPanel

```
public StepTwoPanel()
```


**Method Detail**

### next

```
public WizardPanel next()
```

:   **Description copied from class: `WizardPanel`**
:   Get the next panel to go to.

    :   **Specified by:**: `next` in class `WizardPanel`

---


### display

```
public void display()
```

:   **Description copied from class: `WizardPanel`**
:   Called when the panel is set.

    :   **Specified by:**: `display` in class `WizardPanel`

---


### canFinish

```
public boolean canFinish()
```

:   **Description copied from class: `WizardPanel`**
:   Can this panel finish the wizard?

    :   **Specified by:**: `canFinish` in class `WizardPanel`
    :   **Returns:**: true if this panel can finish the wizard.

---


### validateNext

```
public boolean validateNext(java.util.List list)
```

:   **Description copied from class: `WizardPanel`**
:   Called to validate the panel before moving to next panel.

    :   **Specified by:**: `validateNext` in class `WizardPanel`
    :   **Parameters:**: `list` - a List of error messages to be displayed. **Returns:**: true if the panel is valid,

---


### finish

```
public void finish()
```

:   **Description copied from class: `WizardPanel`**
:   Handle finishing the wizard.

    :   **Specified by:**: `finish` in class `WizardPanel`

---


### validateFinish

```
public boolean validateFinish(java.util.List list)
```

:   **Description copied from class: `WizardPanel`**
:   Called to validate the panel before finishing the wizard. Should
    return false if canFinish returns false.

    :   **Specified by:**: `validateFinish` in class `WizardPanel`
    :   **Parameters:**: `list` - a List of error messages to be displayed. **Returns:**: true if it is valid for this wizard to finish.

---


### hasNext

```
public boolean hasNext()
```

:   **Description copied from class: `WizardPanel`**
:   Is there be a next panel?

    :   **Specified by:**: `hasNext` in class `WizardPanel`
    :   **Returns:**: true if there is a panel to move to next


---


|  |  |  |  |
| --- | --- | --- | --- |
| **Overview** \| **Package** \| **Class** \| **Use** \| **Tree** \| **Deprecated** \| **Index** \| **Help** | | |  |
| **PREV CLASS**   NEXT CLASS | **FRAMES**    **NO FRAMES**     **All Classes** |  |  |
| SUMMARY: NESTED \| FIELD \| CONSTR \| METHOD | DETAIL: FIELD \| CONSTR \| METHOD |  |  |


---
